# COVID-19 outbreak control strategies and their impact on the provision of essential health services in Ghana: An exploratory-sequential study

**DOI:** 10.1371/journal.pone.0279528

**Published:** 2023-11-16

**Authors:** Duah Dwomoh, Isaac Yeboah, Rawlance Ndejjo, Steven Ndugwa Kabwama, Justice Moses Aheto, Anne Liu, Siobhan Lazenby, Fidelia Ohemeng, Sylvia Akpene Takyi, Ibrahim Issah, Serwaa Akoto Bawuah, Rhoda K. Wanyenze, Julius Fobil

**Affiliations:** 1 Department of Biostatistics, School of Public Health, University of Ghana, Accra, Ghana; 2 Institute of Work, Employment and Society, University of Professional Studies, Accra, Ghana; 3 Department of Disease Control and Environmental Health, School of Public Health, Makerere University, Kampala, Uganda; 4 Department of Community Health and Behavioural Sciences, School of Public Health, Makerere University, Kampala, Uganda; 5 Gates Ventures, Kirkland, Washington, United States of America; 6 Department of Sociology, School of Humanities, University of Ghana, Accra, Ghana; 7 Department of Biological, Environmental, and Occupational Health, School of Public Health, University of Ghana, Accra, Ghana; University for Development Studies, GHANA

## Abstract

**Background:**

The COVID-19 pandemic has led to substantial interruptions in critical health services, with 90% of countries reporting interruptions in routine vaccinations, maternal health care and chronic disease management. The use of non-pharmaceutical interventions (NPIs) such as lockdowns and self-isolation had implications on the provision of essential health services (EHS). We investigated exemplary COVID-19 outbreak control strategies and explored the extent to which the adoption of these NPIs affected the provision of EHS including immunization coverage and facility-based deliveries. Finally, we document core health system strategies and practices adopted to maintain EHS during the early phase of the pandemic.

**Methods:**

This study used an explanatory sequential study design. First, we utilized data from routine health management information systems to quantify the impact of the pandemic on the provision of EHS using interrupted time series models. Second, we explored exemplary strategies and health system initiatives that were adopted to prevent the spread of COVID-19 infections while maintaining the provision of EHS using in-depth interviews with key informants including policymakers and healthcare providers.

**Results:**

The COVID-19 pandemic and the interventions that were implemented disrupted the provision of EHS. In the first month of the COVID-19 pandemic, Oral Polio and pentavalent vaccination coverage reduced by 15.2% [95% CI = -22.61, -7.87, p<0.001] and 12.4% [95% CI = 17.68, -7.13; p<0.001] respectively. The exemplary strategies adopted in maintaining the provision of EHS while also responding to the spread of infections include the development of new policy guidelines that were disseminated with modified service delivery models, new treatment and prevention guidelines, the use of telemedicine and medical drones to provide EHS and facilitate rapid testing of suspected cases.

**Conclusion:**

The implementation of different NPIs during the peak phase of the pandemic disrupted the provision of EHS. However, the Ministry of Health leveraged the resilient health system and deployed efficient, all-inclusive, and integrated infectious disease management and infection prevention control strategies to maintain the provision of EHS while responding to the spread of infections.

## Introduction

The severe acute respiratory syndrome coronavirus 2 (SARS-CoV-2), the coronavirus that causes coronavirus disease 2019 (COVID-19) led to an unprecedented number of deaths and morbidities across the globe [1]. Ghana recorded its index COVID-19 case on the 12th of March 2020 and as of the 20^th^ of October 2022, the country has recorded a total of 170321 cases and 1460 COVID-19-induced deaths [2]. Governments across the globe implemented several non-pharmaceutical interventions (NPIs) and vaccination strategies to reduce the burden of the pandemic on health systems and prevent new infections and deaths and increase access to essential health services (EHS). EHS include services with high priority guided by a country’s health system and burden of disease aimed at preventing communicable diseases, reducing maternal mortality and child morbidity, preventing acute chronic conditions, and managing emergency conditions. It focuses on high-priority service delivery as listed in the WHO guidance and tools manual [3]. It includes prevention of communicable diseases, services related to reproductive health (care during pregnancy and childbirth), and care for vulnerable populations such as infants and adults. The high-priority service delivery also includes the provision of medications, continuity of critical inpatient therapies, and auxiliary services such as laboratory services and blood bank services to patients. These NPIs disrupted EHS and compounded the social-economic effects on the populace which made them unsustainable in the long term [4]. As COVID-19 spread, the provision of EHS was negatively affected due to weak health systems, limited-health service infrastructure and poor health financing systems [5]. EHS are disrupted because of the unavailability of health workers, infection of health workers and shortage of essential drugs [6, 7]. Other supply dynamics that contribute to the decline in the use of EHS are lockdown policies and stay-at-home orders [8, 9]. The clients’ fear of contracting the infectious disease results in a decrease in the use of EHS [10]. Understanding how a broader range of NPIs have been instituted to mitigate against the spread of COVID-19, as well as any unexpected adverse effects on EHS demand is essential for future health system planning and pandemic preparedness. Few studies have investigated infection prevention strategies, the impact of COVID-19 on EHS and exemplary interventions implemented to improve the uptake of EHS during the peak phase of the pandemic. We provide evidence of the effect of the pandemic, identify and highlight the best practices adopted by Ghana in maintaining the provision of EHS while also responding to the COVID-19 pandemic using different infection prevention control strategies. The present study is underpinned by the statistical and public health and behavioral change interventions that evaluates health systems’ resilience to fight the pandemic vis a vis the implementation of policies to increase the use and delivery of EHS.

## Methods

This study used an explanatory sequential design in which we collected and analyzed time series data on EHS and then followed the results up with a qualitative phase of in-depth interviews with key informants among healthcare workers and policymakers to explore mitigation strategies adopted to curb the spread of infection and maintain the provision of EHS.

### Study sites

The quantitative study was conducted across the 16 administrative regions of Ghana between March 2020 to December 2021. However, the secondary data analysis of EHS started in January 2018 to December 2021 for all health facilities that report monthly data to Ghana Health Service using the routine health management information system.

### Quantitative study design

#### Data source for interrupted time series analysis

This was a retrospective ecological time series study design using routine time series data on EHS between January 2018 and December 2021. This period was identified as the peak phase of the COVID-19 infection in Ghana. The EHS data was a continuous sequence of observations at the district level taken repeatedly over equal monthly intervals across the 16 administrative regions of Ghana. Interrupted Time Series (ITS) is a quasi-experimental study design with a potentially high degree of internal validity [11]. In this ITS study, a time series of the EHS outcomes of interest were used to establish an underlying trend in EHS utilization, which was assumed to be interrupted by the COVID-19 pandemic on the 12^th^ of March 2020. The ITS was used to model a hypothetical counterfactual scenario under which we assume that had COVID-19 not taken place, the trend on the provision and use of EHS would have continued unchanged (that is: the expected trend, in the absence of COVID, given the pre-existing trend). This counterfactual scenario provides a comparison for the evaluation of the impact of COVID-19 on EHS by examining any change occurring in the period after the first cases of COVID-19 were recorded in Ghana on March 12, 2020. We hypothesized a gradual effect of COVID-19 on EHS (that is, one month lag period before any observed effect).

#### Outcome measures

The study analyzed eight EHS indicators: Antenatal care attendance, facility delivery, post-natal care attendance, oral polio vaccination, Bacille Calmette-Guerin (BCG) vaccination, Pentavalent vaccination, Measles-Rubella vaccination, and outpatients’ visits per 1000 populations (Table 1).

**Table 1:** Outcome indicator definitions.

Indicator	Definition	Numerator	Denominator	Source of denominator
BCG Coverage	The proportion of children under 1 year receiving BCG vaccine	Number of children under 1 year receiving the BCG vaccine in the period	Number of children under 1 year (estimated as 4% of the population)	Ghana Statistical Service from the 2010 Population and Housing Census
Oral Polio vaccination	The proportion of children under 1-year receiving oral polio (OPV1) vaccine	Number of children under 1 year receiving the OPV1 vaccine in the period	Number of children under 1 year (estimated as 4% of the population)	Ghana Statistical Service from the 2010 Population and Housing Census
Pentavalent vaccination	The proportion of children under 1 year receiving Penta1 vaccine	Number of children under 1 year receiving the Penta 1 vaccine in the period	Number of children under 1 year (estimated as 4% of the population)	Ghana Statistical Service from the 2010 Population and Housing Census
Measles-Rubella Coverage	The proportion of children under 1 year receiving Measles-Rubella Vaccine	Number of children under 1 year receiving the Measles-Rubella vaccine in the period	Number of children under 1 year (estimated as 4% of the population)	Ghana Statistical Service from the 2010 Population and Housing Census
Antenatal Care Coverage	The proportion of pregnant women receiving antenatal care during pregnancy (at least once).	Total number of antenatal registrants in a specified period	Total number of expected pregnancies of the catchment area within the specified period	Ghana Statistical Service from the 2010 Population and Housing Census
Skilled delivery	Percentage of deliveries conducted by skilled attendants (nurses and doctors).	The number of deliveries supervised by doctors or nurses in the specified period.	Number of expected pregnancies (estimated as 4% of the population)	Ghana Statistical Service from the 2010 Population and Housing Census
Post-natal coverage	The proportion of PNC registrants seen after delivery	Number of PNC registrants (within 48 hours)	Number of expected pregnancies (estimated as 4% of the population)	Ghana Statistical Service from the 2010 Population and Housing Census
OPD attendance per 1000 population	The rate of facility visits per 1000 population	The number of OPD attendance per month for the index year	Total population per month for the index year	Ghana Statistical Service from the 2010 Population and Housing Census

Source: District Health Management Information System: Ghana Health Service

### Statistical analysis

Descriptive analysis using mean, standard deviation, minimum and maximum observations, median and interquartile range, and tools from time series were used to explore the distribution of the outcome measures and identified the underlying trend, seasonal patterns, and outliers in the EHS data. We tested the normality of all coverage indicators using the Jarque-Bera skewness-kurtosis test (Table 2).

**Table 2:** Skewness and kurtosis test of normality.

	Skewness and kurtosis test of normality
Essential Health Services	*χ*^2^; p-value
Antenatal care attendance	2.09; 0.3516
Facility delivery	5.21; 0.0738
Post-natal care attendance	5.98; 0.0503
Oral polio-vaccination 1	2.99; 0.2247
Bacille Calmette-Guerin (BCG) vaccination	5.15; 0.0762
Pentavalent vaccine 1	1.03; 0.5970
Measles-Rubella-less than 1 month	2.73; 0.2556
Outpatients’ visits per 1000 population	3.61; 0.1641

Our ITS analysis involved three different statistical models. These models were fitted to EHS data before and during COVID. We fitted ordinary least square regression models to quantify the impact of COVID-19 on the provision of EHS. We used the Fourier terms (pairs of sine and cosine functions) to adjust for seasonality and other long-term trends. The following segmented harmonic ITS regression model with Newey West standard errors that adjusts for seasonality in EHS time-series dependent outcomes with sine/cosine pair was used:

$$Y_{ti}=\beta_{0}+\beta_{1}T+\beta_{2}C+\beta_{3}CT+\beta_{4}sin\left(2\pi \omega t_{i}\right)+\beta_{5}cos\left(2\pi \omega t_{i}\right)+\beta_{6}\left(lagY_{ti}\right)+\varepsilon_{ti}$$

*Y*_*ti*_ is the coverage of EHS in the *t*th month of the *i*th year; *t* values range from 1 to 12; *i* values range from 1 to *K*, where *K* is the number of years under observation (2018–2021). *C* is a dummy variable indicating observation collected before COVID (C = 0) or after (C = 1). *T* is a continuous variable that indicates the time in months passed from the start of the observational period (2018–2021).

*β*_0_ represents the baseline level of EHS at *T* = 0, *β*_1_ is interpreted as the change in EHS associated with a unit increase in time and it represents the underlying pre-COVID-19 trend), *β*_2_ is the level change in EHS after the index case of COVID-19 was recorded on 12^th^ March 2020 and *β*_3_ indicates the slope change following the COVID-19 pandemic. *ε*_*ti*_ are independently and identically distributed normal random variables with *E*[*ε*_*ti*_] = 0 and *Var*[*ε*_*ti*_] = *σ*^2^. *ω* reflects the period within a year timespan and in our case, $\omega =\frac{1}{12}$ to reflect monthly data. Our proposed model describes the seasonal behaviour of EHS by both sine and cosine functions with symmetric rise and fall over a period of a full year. For both the Generalized Poisson and the negative binomial regression models, which are required for modeling under and over-dispersed EHS outcomes respectively, we included total OPD cases as an offset variable to convert the EHS outcomes into a rate and adjust for any potential changes in the OPD attendance over time. We estimated the counterfactual by assuming that no COVID-19 case was recorded in Ghana and there was no immediate nor sustained effect of the pandemic. We graphically assessed autocorrelation by examining the plot of residuals and the partial autocorrelation function. This was followed by the Cumby–Huizinga (Breusch-Godfrey) general test for autocorrelation [12]. We employed the Newey-West method [13] to generate more robust standard errors that were valid even when there is the presence of heteroscedasticity and autocorrelation.

### Data sources and Geospatial methods for regional distribution of cumulative confirmed cases

We obtained the number of cumulative confirmed COVID-19 cases from the Ghana Health Service website (https://www.ghs.gov.gh/covid19/archive.php) for the period of 12th March 2020 to 31st December 2021. The regional shapefile for Ghana was downloaded from GADM (https://gadm.org/download_country.html). The rgdal, leaflet and tmap packages in *R* software version 4.1.1 and RStudio were used for Geospatial data preparation [14].

### Qualitative study design

Drawing on public health interventions particularly institutional measures, and behaviour change measures relied on strategies employed for the pandemic control. The pubic health institutional measures include acts and regulations, public investment and subsidies, voluntary standards and guidelines and information and education. The behaviour change measures also include restriction and coercion, persuasion and incentivization, education and training, modeling, enablement and environmental restructuring. The study employed descriptive narrative approach based on the premise that disrupted EHS, barriers and strategies to maintaining EHS and infection prevention control is best understood from the experiences of frontline health workers, policymakers and implementers of policies and programs. This descriptive exploratory study had a qualitative design using a semi-structured interview guide. Frontline health workers shared their experiences with how they controlled the spread of infections while providing EHS. On the other hand, policymakers shared their experiences on policy initiatives and knowledge about how they reduce the spread of infections while maintaining EHS using the narrative approach. A thematic analysis was used as a tool to identify key themes emerging from the interviews. A deductive approach was chosen to identify pre-existing categories or theories by using the experiences and perspectives of frontline healthcare providers and policymakers.

### Participants recruitment

Individuals were considered eligible if they were frontline health workers, policymakers and stakeholders who worked during the COVID-19 pandemic in regions with high recorded COVID-19 cases. Participants were recruited using purposive sampling based on the responsibilities that were assigned to these individuals during the peak phase of the pandemic. The study purposively selected healthcare providers, policymakers, and program implementers. For clarity, health workers who worked in health facilities during the early phase of the COVID-19 pandemic were referred to as frontline health workers and were recruited. Policymakers, implementers and stakeholders who were directly involved with decision-making at the community, district, regional and national levels regarding COVID-19 were also recruited. The study population was among participants in regions with the least and most recorded COVID-19 cases. Hence, the study focused on targeted populations in the Greater Accra, Ashanti, Western, Oti, and Northern regions. Greater Accra, Ashanti and Western regions represent regions that recorded high cases of COVID-19 disease while Oti and Northern regions characterize regions that had low cases of COVID-19 disease. In-depth interviews (IDIs) were conducted among frontline health workers while key informant interviews (KIIs) were conducted among policymakers and implementers. IDIs of frontline health workers were conducted in Greater Accra, Ashanti, Northern and Oti regions. KIIs were conducted in Greater Accra, Western and Ashanti regions. Interviews were conducted between March and May 2022. Potential participants were asked if interested in participating in the study before interviews were conducted. Informed consent was obtained before the interview was conducted by the research assistants. A total of 32 participants (20 frontline health workers and 12 policymakers, implementers, and stakeholders) were involved in this study.

### Data collection

The purpose and significance of the study were communicated to the study participants and interviews were scheduled at the convenience of the study participants. Frontline health workers were recruited from primary, secondary, tertiary, and quaternary health facilities. Policymakers, implementers, and stakeholders were also recruited from the national COVID-19 taskforce, regional rapid response team, municipal/district rapid response team, Ghana Health Service, Municipal health directorate and district health directorate (Detailed background information is provided on Tables 3 & 4). Two different interview guides were prepared before conducting interviews: one each for frontline health workers and policymakers, implementers and stakeholders. The interview guide for frontline health workers consists of contents such as services disrupted, challenges to the utilization of EHS and facility strategies to provide EHS. The interview guide for policymakers, implementers and stakeholders elicited information on disruptions of EHS, challenges to maintaining/restarting EHS and health system interventions to maintaining or restarting EHS. All the interview guides were in English. In addition, all interviews were conducted in English with most of the interviews at the office of the selected participants. Physical distance was maintained during face-to-face interviews while both the research assistant and research participant wore a face mask. All interviews were done on a face-to-face basis. Interviews lasted one hour to one hour and 30 minutes.

**Table 3:** Detailed profile of policymakers/implementers/stakeholders.

Participant ID	Institution	Profession/ Specialty	Level of Team	Region
KII1	Korle-Bu Teaching Hospital	Biomedical Scientist	National Case Management Team	Greater Accra
KII2	Noguchi Memorial Institute for Medical Research	Epidemiologist	National Case Management Team	Greater Accra
KII3	District Health Directorate	Public Health Specialist	District Rapid Response Team	Western Region
KII4	Municipal Assembly	Government Appointee	Municipal/Regional Rapid Response Team	Western Region
KII5	Municipal Health Directorate	Public Health Specialist	Municipal/Regional Rapid Response Team	Western Region
KII6	Municipal Health Directorate	Public Health Specialist	Municipal/Regional Rapid Response Team	Western Region
KII7	Ghana Health Service–Public Health Division	Medical Doctor	National COVID-19 Taskforce	Greater Accra
KII8	Presidential Health Advisory	Medical Doctor	National COVID-19 Taskforce	Greater Accra
KII9	Noguchi Memorial Institute for Medical Research	Virologist	National Case Management Team	Greater Accra
KII10	Metropolitan Health Directorate–Ghana Health Service	Public Health Specialist	Regional Response Team	Ashanti Region
KII11	Metropolitan Health Directorate–Ghana Health Service	Medical Doctor	Regional Response Team	Ashanti Region
KII12	Metropolitan Health Directorate–Ghana Health Service	Public Health Specialist	Regional Response Team	Ashanti Region

**Table 4:** Detailed profile of frontline health workers.

Participant ID	Age	Unit/Specialty	Level of facility	Marital Status	No. of living Children	Region
IDI1	41	Nurse	Tertiary	Married	2	Northern Region
IDI2		Head of Microbiology Department	Tertiary	Married		Greater Accra Region
IDI3	46	Head of Disease Control	Tertiary	Married	4	Ashanti Region
IDI4	60	IPC consultant	Tertiary	Married	4	Ashanti Region
IDI5	48	Nurse	Tertiary	Married	3	
IDI6	53	Principal Nursing Officer	Tertiary	Married	3	Greater Accra
IDI7		Disease Control Officer	Tertiary	Married	2	Greater Accra
IDI8	42	Head of laboratory	Quaternary	Separately	2	Greater Accra
IDI9	36	Midwife	Quaternary	Not married	None	Greater Accra
IDI10	35–45	Emergency Unit	Tertiary	Not married	None	
IDI11	25	Pharmacist	Quaternary	Not married	None	Greater Accra
IDI12	38	Medical Doctor	Tertiary	Married	3	Northern Region
IDI13	37	Nurse manager, Accident and Emergency Department	Tertiary	Married	2	Northern Region
IDI14	45	Pharmacist	Secondary	Married	2	Northern
IDI15	40	Disease Control Officer	Secondary	Married	3	Oti Region
IDI16	34	Medical Laboratory Scientist	Private Tertiary	Married	2	Oti Region
IDI17	32	Nutrition Officer	Private Tertiary	Married	2	Oti Region
IDI18	37	Medical Director	Private Tertiary	Married	2	Oti Region
IDI19	40	Pharmacist	Tertiary	Married	2	Oti Region
IDI20	35	IPC Dept	Tertiary	Married	2	Greater Accra

### Rigour and trustworthiness

The study findings were replicable due to the detailed description of the study methodology and technique. After the interview and transcription, research assistants provided participants with their transcripts as a form of member checking. We did this to establish credibility by confirming that the transcripts reflected the perspectives of the participants interviewed. No research participant raised concerns about the content and quality of the interviews conducted in articulating their perspectives. Research assistants wrote field notes on a daily basis. The field notes include nonverbal indications and interviewers’ reflections. The interviewers had a minimum of 1^st^ degree and substantial experience in conducting IDIs and KIIs. The interviewers do not work in the study settings and had no direct relationship with the research participants. Interview transcripts were shared with authors to review and provide feedback. The daily feedback was done until saturation was reached. Detailed background information about the study is provided below;

### Qualitative data analysis

All interviews were audio-recorded, transcribed verbatim, exported to NVivo (Version 10) and analyzed using the six steps of thematic analysis described by Braun & Clarke [15]. The six steps of thematic analysis involve (1) data familiarization, (2) generating initial codes (3) identifying preliminary themes (4) reviewing themes (5) defining and naming themes; (6) reporting. First, the entire transcripts were read for an overall understanding of the data. Secondly, codes addressing research questions were identified. Thirdly, the initial codes identified were synthesized into wider themes. IY coded the transcripts. The themes from frontline health workers and policymakers and implementers were reviewed and synthesized. In the fifth stage, representative quotes were identified for each theme.

### Ethical considerations

The qualitative and quantitative studies received approval from the Ghana Health Service Ethical Review Committee (GHS-ERC) with a unique approval number GHS-ERC: 008/11/21.

## Results

### Geospatial distribution of cumulative confirmed cases by region

**Fig 1:**
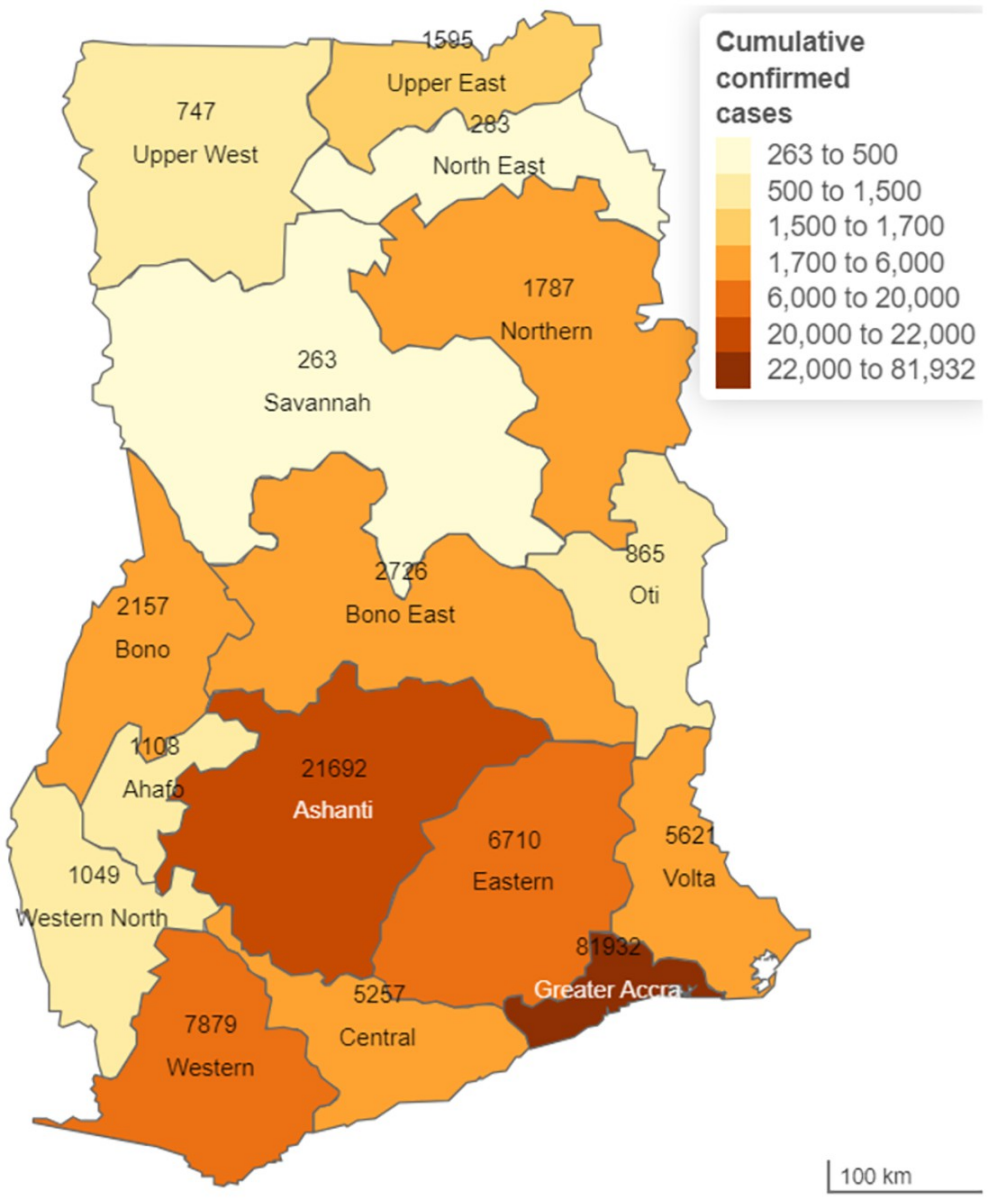
Geospatial distribution of cumulative confirmed cases of COVID-19 by regions in Ghana (12^th^ March 2020 to 31^st^ December 2021).

Our results indicate substantial regional variations in the number of cumulative confirmed cases in the country from 12th March 2020 to 31^st^ December 2021. The Greater Accra region recorded the highest number of cases (81932) (Fig 1).

### Impact of COVID-19 on essential health service delivery

**Fig 2:**
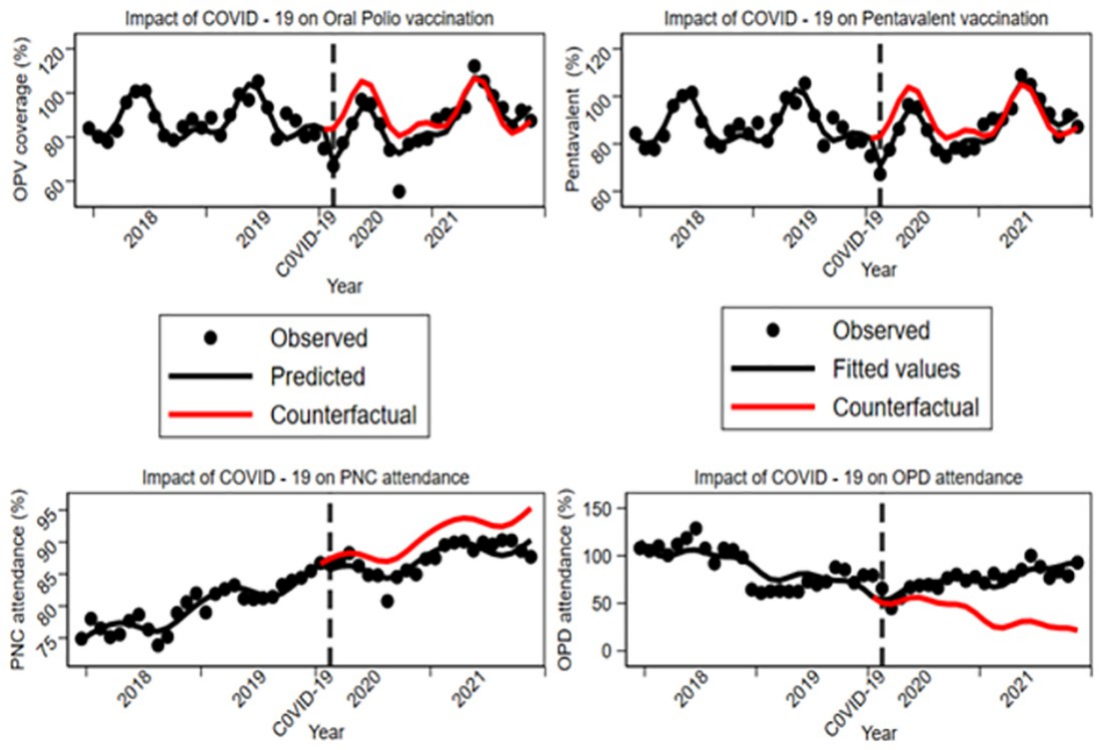
Impact of COVID-19 pandemic on the provision of essential health services.

Table 5 shows the impact of COVID-19 on EHS delivery in Ghana. The pandemic had an immediate effect on BCG, OPV, Measles and Pentavalent vaccination as the coverages of these indicators declined within one month after Ghana recorded its first two index cases. However, it was observed that the pandemic had an immediate negative effect on the provision of EHS (OPV and Pentavalent vaccination coverages) as they declined within the first month of the pandemic. However, the decline in the provision of EHS was not sustained as utilization of EHS began to increase after the first few months of the pandemic. In the years prior to Ghana recording the first two index cases (1^st^ January 2018 to 11^th^ March 2020), OPV coverage was estimated at 88.3% [95% CI: 85.67, 91.26]. In the first month of the COVID-19 pandemic, OPV coverage decreased by 15.2% [95% CI = -22.61, -7.87, p<0.001] which corresponds to the immediate effect of the pandemic. However, this decline was not sustained as the monthly trend of OPV coverage relative to the pre-COVID trend increased by 1.04% on average per month [95% CI: 0.62, 1.46, p<0.001]. Our results on the post-COVID trend analysis showed that after 12^th^ March 2020, OPV coverage increased monthly at a rate of 1.2% [95% CI: 0.80, 1.50; p<0.001]. In the first month of the COVID-19 pandemic, Pentavalent vaccination coverage decreased by 12.4% [95% CI = 17.68, -7.13; p<0.001] which corresponds to the immediate effect of the pandemic. Like OPV, this reduction in coverage was not sustained as the monthly trend of Pentavalent vaccination coverage increased thereafter relative to the pre-COVID trend by 0.9% on average per month [95% CI: 0.53, 1.22; p<0.001]. Our results on the post-COVID trend analysis showed that after 12^th^ March 2020, Pentavalent vaccination coverage increased monthly at a rate of 1.0% [95% CI: 0.72, 1.21%; p<0.001]. PNC attendance decreased immediately after the country recorded the first two index cases, but general OPD attendance has increased since the inception of COVID-19 in Ghana (Fig 2).

**Table 5:** Impact of COVID-19 on essential health service delivery.

	Baseline coverage	*β*_1_ is the slope or trend of the outcome variable until COVID-19 was first recorded in Accra.	*β*_2_ represents the change in the level of the outcome that occurs in the period immediately following Ghana recording the first two index cases of COVID	*β*_3_ represents the difference between pre-COVID and post-COVID slopes of the outcome (effect of COVID-19 over time)	Post-COVID trend, *β*_1_ + *β*_3_	# of Lags
Service utilization	% [95% CI]	*β*_1_[95% *CI*]	*β*_2_[95% *CI*]	*β*_3_[95% *CI*]	(*β*_1_+*β*_3_) [95% *CI*]	
BCG	88.33 [85.67, 91.26]	-0.14 [-0.33, 0.05]	-2.23 [-6.91, 2.44]	0.53 [0.17, 0.89]**	0.39 [0.08, 0.70]*	0
OPV	86.51 [83.62, 89.39]	0.11 [-0.18, 0.40]	-15.24 [-22.61, -7.87]***	1.04 [0.62, 1.46]***	1.15 [0.80, 1.50]***	0
Measles	103.20 [93.70, 112.71]	0.03 [-0.53, 0.59]	-11.15 [-24.64, 2.35]	0.35 [-0.72, 1.41]	0.38 [-0.49, 1.24]	6
Pentavalent	86.87 [84.17, 89.59]	0.09 [-0.17, 0.35]	-12.40 [-17.68, -7.13]***	0.88 [0.53, 1.22]***	0.97 [0.72, 1.21]***	0
ANC	88.99 [85.52, 92.46]	-0.48 [-0.74, -0.23]***	3.19 [-5.20, 11.58]	0.58 [0.05, 1.12]*	0.10 [-0.43, 0.63]	2
Skilled delivery	61.88 [58.36, 65.39]	-0.15 [-0.38, 0.08]	-2.15 [-7.89, 3.60]	0.65 [0.29, 1.00]**	0.50 [0.13, 0.87]**	8
PNC	74.21 [73.46, 74.96]	0.46 [0.41, 0.50]***	-1.66 [-3.31, 0.00]	-0.16 [-0.28, -0.03]*	0.30 [0.18, 0.41]***	7
OPD attendance per 1000 population	114.23 [101.18, 127.29]	-2.08 [-3.17, -1.00]***	2.67 [-19.99, 25.34]	3.25 [2.13, 4.38]***	1.17 [0.52, 1.82]**	8

p-value notation:

***p<0.001,

**p<0.01,

*p<0.05

### COVID-19 outbreak control strategies

The results from the key informant interviews showed public health institutional measures categorized into five themes adopted to mitigate the impact of COVID-19 in Ghana: (1) rapid response coordination with preparedness & response plan leading to a reduced number of new cases and deaths: swift partial lockdown in hardest-hit regions (Greater Accra and Kumasi in the Ashanti region), (2) the adoption of pre-existing surveillance systems, leveraging previous experiences for managing epidemics; supplemented by community serological surveys provided real-time data for decision-making, (3) the implementation of 3T’s strategy: test, trace, and treat; swift contact tracing & isolation measures in early phases of the pandemic, ramp up testing through various strategies, leveraging existing GeneXpert lab equipment, pooled testing, improved vaccination strategy, private sector engagement, mass testing in hotspots, and early RDT adoption contributed to limiting the spread of the disease and preventing deaths. (4) Sustained multisectoral coordination mechanisms with political leadership commitment, including academia, donors, and private sector; mirrored at regional and district level increased the production of PPEs and(5) public education.

#### Theme 1: Rapid response coordination

Participants observed governments commitment and swiftness in rapid response coordination manifested as interventions such as private sector collaboration, legalization of non-pharmaceutical interventions, training of health personnel about the COVID-19 disease, and construction of laboratory sites.

A participant highlighted the role of private sector groups in the provision of hand sanitizers and other essential services. According to a participant, the President of Ghana met diverse groups including pharmaceutical companies. These companies were innovative to ensure that the local market produced affordable hand sanitizer.

*“His Excellency the President met the various private sector groups*, *especially the private pharmaceutical companies*, *[and] within a matter of 48 hours*, *about 37 different companies put one of their lines to produce [hand] sanitizers*. *And you remember that [hand] sanitizer was a very essential commodity*. *Within a matter of about 2 to 3 weeks*, *it became something everybody could afford*, *and the price went down considerably*.*”*

*(KII2)*

Some participants mentioned that the ability of the government to build more laboratory testing sites helped in the fight against the COVID-19 pandemic in Ghana. The government was able to build on the existing few laboratory sites with the support of the private sector. Prior to covid-19 pandemic, Ghana had only two laboratory sites. However as at time of data collection, a policy maker indicated that the country now have 45 laboratory sites.

“*When we started*, *they were only two*. *[Things began to change] when the government then allowed that the private sector and the rights protection join in this fight*, *and*, *within a matter of months*, *we now had about 45 laboratory testing sites*. *More than half of them were private-owned*.*”*

*(KII2)*

Participants were trained on infection, prevention, and control of the COVID-19 disease. The trainings content include case management training, contact tracing and surveillance. Particiapnts further revealed that the traning helped them to stick to infection, prevention and control measures.

*“Staff commitment to training on IPC and their readiness to stick to infection prevention control measures really helped us*.*”*

(IDI2)

*That was when*, *you know*, *national training actually kicked started*, *even though some prior stakeholder engagement had taken place*, *but it had to take the announcement of those cases for case management training*, *contact tracing*, *surveillance*, *in terms of their whole response*. *And national’s trainer of training began after we had announced our cases*. *So*, *yes*, *it shook us*, *but then*, *I think that we began responding in doing all the things that had to be done*

*(KII12)*

Participants agreed that government NPIs, backed by legal legislation, helped to control the spread of COVID-19. One of such NPIs is the closure of land, air, and sea borders.

*“I think one important thing that we did that most countries didn’t do is the limitation of the importation of business by closing all our borders*. *Luckily*, *we have only one [international] airport*, *so [it was easy to see at first hand the cases that were being imported]*.*”*

*(KII2)*

Participants indicated adherence to COVID-19 protocols by both patients and healthcare providers. At the early stages of the pandemic personal protective equipment such as face masks were not wore very well. However, the healthcare professionals ensured that their fellow healthcare professionals strictly adhered to wearing of gowns, head gear and others.

*“At the height of the pandemic everyone was adherent to wearing masks*, *even though people were wearing the wrong types of masks and were wearing them in various ways when interacting with patients*. *But with regards to gowns*, *head gears*, *and others worn by the team [while] interacting with the patients*, *those were strictly adhered to in my facility*.*”*

(IDI3)

#### Theme 2: Adoption of pre-existing surveillance systems

Participants elaborated on the surveillance systems utilized, such as DHIMS (District Health Information Management System) and SORMAS (Surveillance Outbreak Response Management and Analysis System), which played critical role in gathering precise data. Both DHIMS and SORMAS offer avenues for monitoring and evaluation purposes.

*“We have a platform where*, *on a weekly basis*, *all the district officers on the platform share their planned activities for the week*. *At the regional level too*, *we look at the data that we have on the DHIMS*. *So we identify the districts that have some challenges or need assistance and assist them*. *We also look at the data for priority diseases like epidemic-prone diseases [such as] yellow fever and measles*. *We also download the data on the DHIMS and follow up on the cases reported to verify the numbers*.*”*

(IDI2)

#### Theme 3: Implementation of testing strategies

The testing strategies encompass various types of Covid-19 tests and preventive measures implemented by the case management team. Some of the strategies include 3T (trace, test and treat) and pooling strategy.

A participant noted that healthcare providers used the 3T strategy. This involved testing a patient and when tested positive you do tracing of close relatives or neighbors while the Covid-19 patient is isolated for treatment. This strategy was employed in all testing sites under the supervision of disease control.

*“…[for] the testing strategy…you identify first*, *you test*, *[and] if it’s positive*, *you isolate*. *Then you have to do contact tracing*. *But the basic idea was that the moment you hear of anybody having the symptoms or anybody reporting [to have] symptoms*, *you quickly take their sample [and] test*. *When it’s confirmed*, *you go back [and] do contact tracing while the [sick] person is isolated*. *That was the strategy [that was] communicated to all the testing sites [and] disease control officers*.*”*

*(KII4))*

A participant observed that one of the unique testing strategies employed by Ghana’s COVID-19 data management team was pooling. By pooling, individual samples are tested to save time and cost, as well as reduce the use of test kits.

*“The second one was the method we call pooling*. *The pooling [began] when we had so many contact tracing samples*. *When somebody tested positive*, *we went and collected a lot of samples around*, *and*, *instead of working on individual samples*, *we pooled samples together—sometimes five samples in one or ten samples in one*. *So*, *if you have 100 samples*, *in the end you have only 10 to work with*. *So*, *on a daily basis*, *we were sometimes able to process about 5*,*000 samples*.*”*

*(KII10)*

#### Theme 4: Sustained multisectory coordination

A participant highlighted the ways in which the government’s pre-existing multisectoral coordination strengthens the battle against COVID-19. The national health system is organized into four tiers: community, district, regional and national levels. Each level of health facilities is equipped with prevention and case management unitis to effectively address public health emergencies.

*“The National Health System in place is a bit broad*. *At the Ministry of Health and Ghana Health Service*, *we have facilities at the national*, *regional*, *and district levels*. *At each of these levels there are facilities for prevention and case management to respond to public health emergencies*. *Knowing that the health system and the determinants of health are very broad*, *the system does not end only in the health sector*. *Various components and players and actors whose activities impact on public health are considered as part of the National Health System”*.

*(KII7)*

Further, participants explained the existing emergency preparedness plan used to counter any outbreak in the various facilities.

*“The facility has an emergency preparedness plan for all eventualities and outbreaks*. *They have a plan*, *so if there is an outbreak they use that plan*, *and if there are some*

*changes*, *they incorporate those changes*.*”*

(IDI8)

#### Theme 5: Public engagement

According to a participant, during the peak stage of the pandemic, there was a significant emphasis on intensive public education, while healthcare facilities were simultaneously handling a surge in COVID-19 cases.

*“At the peak stage*, *[public] education [intensified]*. *So*, *people’s fear was not as bad as it used to be*, *even though we were having a lot more cases in our facility.”*

*(IDI18)*

There was regular education by the media regarding the COVID-19 pandemic. Citizens were assured that all precautionary measures were strictly adhered to in the health facilities, hence, an assurance of minimal risk of infection.

*“With the information we heard on radio people began to understand that no matter how we felt*, *we needed to report to a hospital for the hospital to decide whether it was COVID-19 or not*. *[It was safe] to go to the health facilities because all the precautions were observed there*, *and our chances of getting COVID from the health facility were quite minimal*.*”*

(KII6)

### Strategies to maintain essential health services

The behavioral change interventions used in this study are based on an integrative framework developed based on 19 frameworks which was later categorized into 9 interventions. In this study, we categorized these practices adopted to provide into three behavioural change interventions: 1) persuasion and incentivization 2) enablement and environmental restructuring and 3) restriction. Interventions rolled out under persuasion and incentivization include motivating staff, provision of transportation for staff, community engagements and home visits. Enablement and environmental restructuring interventions include patient appointment, non-closure of facility and telemedicine and drone services. Lastly, restriction involves implementing interventions such as triage station and provision of PPEs.

#### Theme 1: Persuasion and incentivisation

This theme involves health workers attempt to convince people that behaviour will result in good outcomes. We found in our study that this theme was achieved through 1) engagements with community and home visits; 2) motivating staff and 3) provision of transportation for staff.

The study revealed that health workers were motivated through the provision of vehicles to convey them to the healh facility to provide health services. Some participants were supported with the provision of buses that took them to their workplace.

*“They made sure there was a driver and a vehicle*. *Any time we took a sample we called the driver to make sure they got the sample to the reference lab*.*”*

(IDI7)

From the study policymakers and implementers explained that health personnel were encouraged to put in their best in the dire moments when the government and the people of Ghana needed them most.

*“As leaders of the health system*, *we were tasked to motivate our staff to be at post*, *because the risk was for all*. *As managers*, *we needed to motivate our people and encourage them—not financially—whenever we could to let them know how important their role was in the management of the COVID-19 outbreak and provision of essential sergices*, *so we did that*.*”*

(KII11)

Participants observed that health workers were moving into the homes of patients to provide them with health services. Home visits were a targeted approach to patients.

*“Within the period*, *antenatal services were not affected much because we used to go to pregnant women in their homes*.*”*

(KII1)

There were community engagements to encourage the local people to attend hospitals for their good.

*“We engaged with the community to let them know that [people could come to the hospital]*. *Initially*, *the communication was that if you were not sick*, *you should not come to the hospital*, *so when things changed*, *we needed to go back to them to let them know that all services were back and running*, *and they could come*.*”*

(KII8)

#### Theme 2: Enablement and environmental restructuring

This theme deals with making the behaviour easy to do with facility interventions. Findings revealed interventions including patient appointment, non-closure of facilities, and telemedicine and drone services. All these interventions were done to facilitate essential health services as well as coping with Covid-19 viral transmission.

Regarding non-closure of health facilities, the health facilities were open for 24 hours to allow patients to visit for any medical need at their convenience time including essential health services. In addition, the public was assured that the health facilities adhere to strict screening to reduce the risk of getting infected at the health facility.

*“As I said*, *we didn’t close any health facility*. *They were running 24/7 as they used to*, *and as part of the education that we gave the people*, *we told them that we were still working*, *so anybody who had healthchallenge could still come to the hospital*. *We even showed them our workflow*, *which included screening everyone entering the facility*, *to assure them that the risk of getting infected at the health facility was low*.*”*

(KII1)

One of the innovative strategies employed by health facilities is the appointment system. By appointment strategy, healthcare professionals scheduled meeting times with patients. The appointment strategy reduced the effects of the inadequate number of frontline health workers in the facilities due to additional tasks and responsibilities resulting from COVID-19-related activities. These were all geared towards providing essential health services while covid-19 transmission is curtailed.

*“The appointment system helped*. *It reduced patients’ wait time to see a doctor*. *COVID-19 taught us to use such a system and reduce the number of trips chronic patients had to make to the hospital for a refill of their medication*. *Our capacity in terms of testing for COVID-19 was lacking*, *but the appointment system helped to regulate visits to testing site s and to also provide other services for our clients*.*”*

(KII12)

Some facilities employed innovative ways of providing health services to their clients. One of such innovative approaches was the use of telemedicine and drone services.

*“We had an app*, *COVID Connect*, *by which people we treated and discharged could keep in contact with us*, *and people with symptoms could go into the app and indicate their symptoms for some healthcare professionals from our facility to contact them*.*”*

(IDI11)

*We made use of it [drones]*, *it assisted us especially when we had very few testing centers*. *We were able to use the Drones to gather all the samples to the Drones Distribution center and send them to where we were having only two testing sites at Korle-Bu*, *Noguchi and ACCR to make use of the Drone system*. *And during the time that we were also doing the vaccination*, *we also made use of the Drone system because the Drone distribution center is like the Medical stores*.

*(KII2)*

#### Theme 3: Restriction

This theme entails enforcing interventions that will reduce the spread of Covid-19, thereby, putting in hope in patients to visit health facilities for essential health services. These include triage station and provision of PPEs.

Facilities instituted a station where patients were screened for Covid-19 before entering the health facilities. Establishing a triage station ensured that there was a designated physical area where frontline health workers assessed and refer patients to reduce transmission of Covid-19 and also ensured patients had the confidence to seek essential health services at the facility.

*“But we quickly also got around that by introducing measures in terms of triaging*, *screening patients”*

(KII5)

*“There was a triaging system put in place where*, *for anyone who came in*, *you ensured first of all that the person did not have COVID-19 before you proceeded to do anything else*. *COVID-19 tests were the first things we did for patients who presented*. *These made patients have the trust to seek other health services”*

(IDI11)

Policymakers and implementers explained that PPEs such as face masks and goggles were distributed across health facilities in Ghana. In addition, health care workers were educated to always put on the right PPEs. This was, however, confirmed by the healthcare professionals.

*“And then every single person in the hospital*, *and of course*, *those going out had to be in the right PPE*, *i*.*e*., *face mask*, *and where possible*, *goggles*.*”*

(KII11)

## Discussion

In this study, we quantified the impact of COVID-19 on the delivery of EHS, explored practices adopted to maintain the provision of EHS, and identified exemplary IPC strategies in Ghana. Our results showed that the pandemic disrupted the provision of EHS especially immunization coverage indicators within the first month of the pandemic similar to what has been reported elsewhere [16, 17]. This study highlights that with the rapid spread of COVID-19, mothers avoided taking their children to health facilities to seek child health services including immunization and vaccinations. This could also be attributed to imposed travel restrictions by the government of Ghana during the early days of the pandemic in March 2020 which affected the physical accessibility of EHS facilities. Findings illustrated that fear and stigma influenced the reduced health care in facilities. This aligns with the study by Al-Zaman [18] which indicated that fear and anxiety about the pandemic among communities affected the utilization of EHS. The inadequate health workforce for COVID-19 surge capacity demand proved as a challenge to the utilization of EHS.

As mentioned earlier, although COVID-19 disrupted EHS during the early phase of the pandemic especially OPV and Pentavalent vaccination among children under five, the impact was not long felt due to the systems that were put in place to maintain the provision of EHS in Ghana. These includes new policy guideline that were developed and disseminated with modified service delivery models, non-closure of health facilities to allow access at all times, use of appointment systems, provision of PPEs, staff motivation for frontline HCWs and lab technicians, home visit to provide EHS, intense public education geared towards encouraging facility visits, use of telemedicine and drone services to supply essential medical equipment’s in situations where transportation was not readily available, use of an appointment system for MCNH services at the facility, effective government communication and commitment to increase access to EHS during the peak phase of the pandemic that resulted in-home visits, and the timely order of logistics to prevent stock out of essential supplies. Literature highlights public health institutional measures and behavioural change interventions to sustain the delivery of EHS. Using behaviour change interventions, positive COVID-19 attitudes and perceptions were promoted through public education and routine home visits.

As a form of public health institution interventions, the study finds that measures adopted include adoption of pre-existing surveillance system, public education, rapid response coordination, implementation of 3T’s–test, trace and treat and sustained multisectoral coordination mechanisms. This inadvertently led to a reduction in the utilization of EHS such as child health services as found in other studies [19]. With the upsurge of Covid-19, building a resilient health system in SSA was a challenge. We found that one of the institutional interventions implemented to reorganize and maintain access to safe and EHS was the screening and establishment of triage in health facilities. The screening and creation of triage of suspected COVID-19 patients reassured them of their safety in the health facilities when they visit. Another institutional intervention used was telemedicine. Using telemedicine approaches such as COVID Connect–an application that keeps patients and health workers in contact proved critical to providing EHS. Among other benefits, this application identifies hotspots, manages and registers attendance at events, tracks government updates and monitors symptoms. Other studies indicated that telemedicine increases trust and fosters feelings of intimacy and relief when health workers and the patient already know each other [20, 21].

As a behavioral change intervention, the study found that the government of Ghana implemented different behavioural change intervention packages to motivate and protect frontline health workers at the forefront of the pandemic fight. The packages include providing them with an allowance of 50% of their basic salary and also making available free buses to and from work at specific routes. Other behavioural change interventions include non-closure of facilities and provision of PPEs in health facilities for frontline health workers. This assured the public of availability and readiness to serve their health needs. The government also distributed PPEs across the country to increase the safety of frontline health workers and patients.

There is a correlation between IPC strategies and the provision of EHS. Adopting effective prevention control measures reduces the burden of the pandemic on health systems paving the way to provide EHS. The integrated interventions implemented by the Government of Ghana and other stakeholders contributed significantly to the minimal impact of the pandemic on the health system and this consequently increased the ability of the health system to provide EHS in the hardest hit regions.

### Strengths and limitations of the study

This remains the only study that has simultaneously explored the complex interplay between the infection outbreak control strategies, the impact of the pandemic on EHS and practices geared towards maintaining the provision of EHS during the early phase of the pandemic. Our study has some limitations. The qualitative data may not be representative of the target population as it largely reflects the opinion of the key informants that were interviewed. That is, caution and contextual consideration must be applied when the intent is to directly transfer the knowledge gained and entire results to other contexts and settings. Although more rigorous statistical methods have been employed to quantify the impact of the pandemic on EHS delivery, our study could suffer from potential selection bias due to the quasi-experimental design and caution must be applied when the intention is to infer causality.

## Conclusion

This study provides evidence of the impact of COVID-19 on the provision of EHS and exemplary outbreak control strategies. Although EHS were disrupted during the early phase of the pandemic, the complex integrated approach that includes infection prevention control strategies, resilient health system, government commitment, private sector and stakeholder engagements contributed significantly to the effective management of the pandemic and the provision of EHS.
